# Clinical validation of 4K three-dimensional exoscope system in stapedotomy: a retrospective cohort study

**DOI:** 10.3389/fsurg.2026.1856818

**Published:** 2026-07-03

**Authors:** Chaoyue Zhao, Bo Gao, Guojian Wang, Honglei Zhang, Xin Zhang, Wei Liu, Dongyi Han, Yongyi Yuan, Hang Shao, Pu Dai

**Affiliations:** 1 Nankai University, Tianjin, China; 2 Senior Department of Otolaryngology Head and Neck Surgery, The 6th Medical Center of Chinese People's Liberation Army General Hospital, Chinese People's Liberation Army Medical School, Beijing, China; 3 State Key Laboratory of Hearing and Balance Science, Beijing, China; 4 National Clinical Research Center for Otolaryngologic Diseases, Beijing, China; 5 Key Laboratory of Hearing Science, Ministry of Education, Beijing, China; 6 Beijing Key Laboratory of Hearing Impairment Prevention and Treatment, Beijing, China; 7 Air Force Medical Center, People's Liberation Army, Beijing, China; 8 Yangtze Delta Region Institute of Tsinghua University, Jiaxing, Chin

**Keywords:** 4K three-dimensional exoscope system, operative microscope video acquisition system, otologic surgery, stapedotomy, surgical ergonomics

## Abstract

**Purpose:**

To evaluate the technical feasibility and ergonomic advantages of 4K three-dimensional exoscope systems (4K3D-ES) versus operative microscope (OM) in stapedotomy.

**Methods:**

A retrospective cohort analysis compared 140 consecutive stapedotomy procedures performed by two senior surgeons (4K3D-ES: *n* = 52 by Surgeon PD; OM: *n* = 88 by Surgeon DYH) at a tertiary referral center. Outcome measures included operative time (total/procedural phases), intraoperative blood loss, pre- and postoperative audiometric results, complication rates, and ergonomics and image quality of operators were evaluated via a questionnaire.

**Results:**

The 4K3D-ES system demonstrated technical feasibility across all stapedotomy procedures, achieving complete procedural success without intraoperative conversion to OM. Analysis of surgical efficiency metrics revealed statistically comparable performance between the two groups: total operative duration showed no significant difference (*p* = 0.204), paralleled by equivalent intraoperative blood loss (*p* = 0.075). Critical procedural phases—including time of exposing the footplate (*p* = 0.05), footplate fenestration and prosthesis placement phases (*p* = 0.207)—showed no significant differences. Pre- and postoperative audiological results proved clinically equivalent, with both cohorts achieving substantial air-bone gap improvement while demonstrating comparable closure ABG (*p* = 0.077). The safety profile proved excellent, with complete absence of major complications such as sensorineural hearing loss, facial nerve or chorda tympani nerve injury. Transient vestibular symptoms occurred in minimal cases (4K3D-ES:2; OM:1), while longitudinal follow-up revealed no delayed complications. Surgical teams' evaluations substantiated the 4K3D-ES's clinical utility through unanimous controllability endorsement, predominant stereoscopic image approval, and widespread ergonomic comfort recognition.

**Conclusion:**

4K3D-ES demonstrates technical equivalence to OM while offering superior ergonomics and team-shared 3D visualization, establishing its viability for precision stapes surgery.

## Introduction

Stapedotomy is an operation used to treat otosclerosis and related diseases of the fixed stapes footplate, and it is usually performed under an operation microscope (OM). The intricate three-dimensional anatomy of the stapes surgery, encompassing critical neurovascular structures and delicate osseous components, necessitates high-fidelity optical magnification for safe surgical navigation (1). Conventional OM has served as the cornerstone of otologic procedures, enabling stereoscopic visualization essential for manipulating submillimeter-scale structures. Their ubiquitous integration into microsurgical practice has substantially improved procedural accuracy while reducing intraoperative risks (2–4).

Traditional OM systems restrict optimal three-dimensional visualization to the primary surgeon, with assistants limited to observation through secondary ports. This paradigm mandates sustained postural alignment with the ocular lenses, inducing localized musculoskeletal strain during prolonged procedures. Furthermore, ancillary personnel—including trainees and scrub nurses—are relegated to interpreting two-dimensional projections, resulting in compromised spatial perception that impedes both intraoperative collaboration and surgical education.

Recent advancements in 4K three-dimensional exoscope systems (4K3D-ES) offer a paradigm shift in intraoperative visualization (5–7). These systems theoretically provide stereoscopic visualization with clear delineation of vascular and structural anatomy. Preliminary evidence suggests ergonomic advantages, particularly in mitigating cervical strain associated with prolonged microscope use (8, 9).

Despite growing interest, 4K3D-ES applications in otology remain confined to isolated case reports and limited procedural evaluations (10). This study compares the technical performance and ergonomic profiles of 4K3D-ES and traditional microscope in stapes surgery, addressing the central question: Can 4K3D-ES overcome the inherent limitations of conventional systems while maintaining surgical precision, thereby establishing itself as a reliable visual alternative in oto-microsurgery?

## Materials and methods

## Study design and group allocation

This retrospective cohort study evaluated stapedotomy procedures performed at the Department of Oto-Lateral Skull Base Surgery, People's Liberation Army (PLA) General Hospital (Beijing, China) between January 2021 and March 2024. The study was not randomized. Group allocation was determined by two factors: the availability of the 4K3D-ES system, which was introduced into clinical practice in January 2020, and the primary surgeon's preferred visualization modality. Patients undergoing stapedotomy for conductive hearing loss or mixed hearing loss consistent with otosclerosis were included.

Surgical interventions were stratified into two cohorts: those utilizing the 4K3D-ES system (AINNOVI, China) and those employing traditional binocular microscope (KINEVO 900, ZEISS, Germany). Patients in the 4K3D-ES group consisted of all consecutive stapedotomy procedures performed by surgeon PD using the 4K3D-ES system between January 2021 and March 2024. Patients in the OM group consisted of all consecutive stapedotomy procedures performed by surgeon DYH using the traditional operative microscope during the same study period (January 2021 to March 2024). Both surgeons are senior neurotologists with >10 years of experience and >3,000 otologic procedures each, and both adhered to the same standardized surgical protocol (Fisch technique). No cases were excluded based on anatomical complexity, surgical difficulty, or anticipated outcomes. Due to the non-randomized design and the involvement of two different surgeons, the results should be interpreted as a comparison of two surgical platforms in real-world clinical practice rather than a direct head-to-head device comparison with complete confounding control.

Patient selection required confirmed otosclerosis diagnosis with preoperative air-bone gap ≥15 dB and pure-tone average ≥35 dB at 500–4,000 Hz. Exclusion criteria encompassed incomplete audiometric evaluations, inadequate surgical video documentation, or deviations from standardized Fisch technique - including endaural approach, laser/microdrill-assisted stapedial tendon sectioning, footplate fenestration, and titanium prosthesis implantation (11). Surgical documentation comprised comprehensive 4K video recordings for all cases, with supplemental stereoscopic still images captured in the 4K3D-ES group. Postoperative audiometric assessments were performed at 2 weeks after surgery, with subsequent follow-up ranging from 0.5 to 12 months. These follow-up assessments evaluated air conduction, bone conduction, and air-bone gap (ABG) at standard frequencies (500–4,000 Hz), facial nerve function, and vestibular symptoms.

The 4K3D-ES was depicted in our previous study (6). Two iterations(first- and second-generation) of the 4K three-dimensional exoscope system (4K3D-ES) were used in this study. The first-generation system consisted of a dual-channel 3,840 × 2,160 resolution exoscope with external video processing workstations and dual 55-inch polarized LCDs. The second-generation system integrated optical acquisition, 3D rendering, and real-time video distribution into a compact microsurgical imaging platform, with foot-pedal controlled stereoscopic capture and multi-viewer synchronized observation. Both generations allowed simultaneous viewing by surgeon and assistant and supported stereoscopic 3D visualization using dual-channel projection. Both configurations provided foot-pedal controlled stereoscopic picture capture, multi-viewer synchronized observation via polarized glasses, and 4K surgical video documentation capabilities. All captured images are in 4K resolution, providing high-resolution images for documentation and teaching purposes. The microscope used in the OM group was a ZEISS KINEVO 900, with an image resolution of 3,840 × 2,160, a working distance of 200–625 mm, and a system latency of 130 ms.

**Figure 1:**
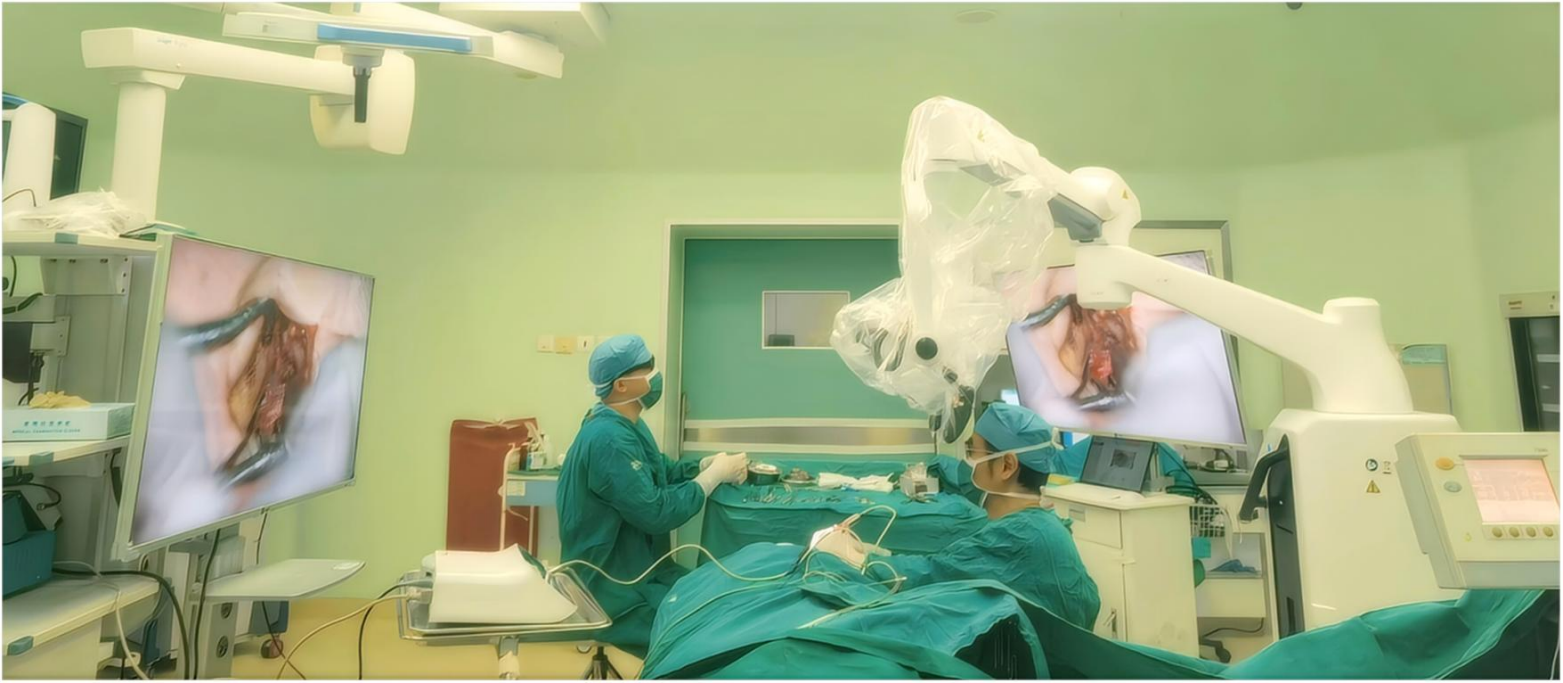
Standard setup in otologic microsurgery. The image shows the operating room set-up, both the trainee and surgeon have the same field of view on the LCD. And the latest generation of 4K3D-ES has been applied in clinical surgery.

Surgical teams performed surgeries through synchronized 4K LCD monitors using passive polarized eyewear, enabling dynamic multi-axial visualization without postural constraints (Figure 1). All stereoscopic image pairs in this investigation were visualized through two distinct modalities: 1. Dual-channel polarized projection (Supplementary Figure 1A): Independent left/right perspective images were delivered to corresponding eyes through two orthogonal polarizations, generating retinal disparity for depth perception; 2. Dual-channel direct projection (Supplementary Figures 1B,C): By using a set of presbyopia spectacles (+6.00D to +7.00D) magnify left/right perspective image to each eye to achieve 3D vision with this system. Both methods engaged primary visual cortex pathways, where disparity-sensitive neurons synthesized 3D perception through interhemispheric signaling.

The user-friendly questionnaire for the 4K3D-ES system was administered to 3 experienced neurotologists and 14 otolaryngology interns immediately following surgery. The questionnaire assessed four domains: system controllability (ease of handling, foot pedal responsiveness, camera maneuverability); image quality (stereoscopic depth perception, color fidelity, resolution); ergonomic comfort (neck/shoulder strain, back discomfort, overall posture); and adverse symptoms (eye fatigue, dizziness, headache). Each item was rated on a 4-point Likert scale: “very good,” “good,” “acceptable,” and “not acceptable,” with results presented in percentage form. Additionally, participants were given the opportunity to provide comments on any concerns not covered by the questionnaire.

The patient demographics, operative time, amount of intraoperative bleeding and user-friendly score were calculated. We performed descriptive statistics and statistical analysis of the collected data to compare 4K3D-ES and OM groups. Continuous variables were expressed as mean ± standard deviation (SD). Normality of distribution was assessed using the Shapiro–Wilk test. For variables that met the normality assumption (operative time phases, intraoperative blood loss), independent Student's t-test was used for between-group comparisons. For ordinal variables (questionnaire ratings) and continuous variables that violated the normality assumption (if any), the Mann–Whitney U test was applied. Categorical variables were analyzed using the *χ*² test or Fisher's exact test as appropriate. All statistical tests were two-tailed, and a *p*-value <0.05 was considered statistically significant.

## Results

Our analysis included 140 stapedotomy patients meeting the strict selection criteria. As shown in Appendix Table 1, the OM group (88 cases) and 4K3D-ES group (52 cases) had remarkably similar baseline profiles—age, gender distribution, and operative side showed no significant differences, setting a solid foundation for comparison. Notably, both cohorts were derived from consecutive surgical series without case selection based on anatomical complexity or anticipated surgical difficulty. None of the patients showed facial nerve malformations during the operation.

**Figure 2:**
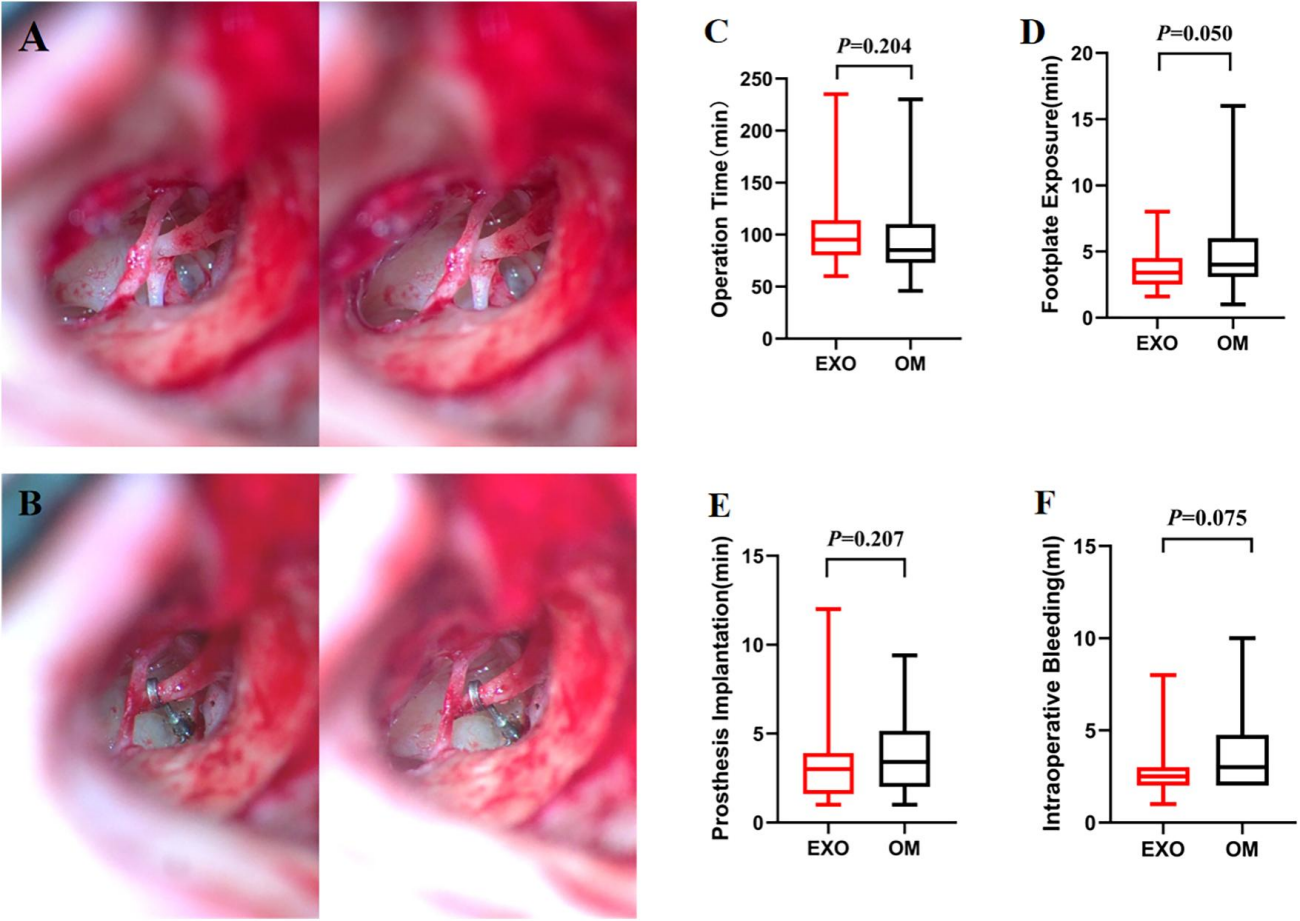
Stereoscopic images of stapedotomy through 4K3D-ES. **(A)** After endaural incision and scutum curettage, the structure of the tympanic chamber is fully exposed. **(B)** After fixation, hook part of the artificial stapes is firmly connected with the incus and does not affect the blood supply of the incus. The comparative evaluation of stapedotomy using 4K3D-ES (red) and OM (black). **(C)** The whole operation time; **(D)** The time for violating the integrity of the incudostapedial joint and of the stapedial arch; **(E)** The time for fenestrating into the footplate and placement of the prosthesis; **(F)** The intraoperative blood loss between 4K3D-ES and OM groups.

Interestingly, when we reviewed surgical recordings (Figures 2A,B) and timed the procedures, both procedures proved equally efficient. Figures 2A,B illustrates operative views for 4K3D-ES and OM. Although quantitative metrics were similar, surgeons reported enhanced depth perception, improved visualization of the stapes footplate and to critical structures with the 4K3D-ES system. The ability for both surgeon and assistant to share a stereoscopic 3D view was highlighted as a qualitative benefit for teaching and intraoperative coordination. Total operative duration (Figure 2C, *p* = 0.204), time of transecting the incudostapedial joint and the stapedial arch (Figure 2D, *p* = 0.05), time of creating a fenestra into the footplate and placement of the prosthesis (Figure 2E, *p* = 0.207), and intraoperative blood loss (Figure 2F, *p* = 0.075) all showed comparable results. This was somewhat surprising given the new technology's learning curve, but clearly demonstrates that 4K3D-ES keeps pace with OM in procedural fluidity. To address potential confounding by surgeon effect and learning curve, we conducted two sensitivity analyses: a subgroup analysis restricted to procedures performed after the first 20 cases in the 4K3D-ES group to minimize the learning curve effect. Operative times were slightly longer during the initial cases, but postoperative hearing outcomes and safety profiles remained consistent with the overall cohort. Inclusion of the first 20 cases in the overall analysis did not significantly alter the results.

Hearing results from pre- and post-operation (0.5–12mo) were analyzed. Preoperative air-conduction thresholds (PTA) and air-bone gaps (ABG) were nearly identical between groups (*p* = 0.492 and *p* = 0.751). At the ≥2-week postoperative check, hearing improvement occurred in both cohorts (Appendix Table 2), with ABG reduction showing no significant difference. Postoperative air-bone gap (ABG) closure rates were analyzed between the 4K3D-ES and OM groups. In the 4K3D-ES group, 31 of 52 patients (59.6%) achieved an ABG <15 dB, whereas in the OM group, 42 of 88 patients (47.7%) achieved the same threshold (*p* = 0.236). Safety records were equally reassuring: no sensorineural hearing loss, facial paralysis, or taste dysfunction occurred. Transient vestibular symptoms (dizziness) occurred in 2 patients in the 4K3D-ES group and 1 patient in the OM group. No patients reported persistent dizziness at the final follow-up (4–38 months).

**Figure 3:**
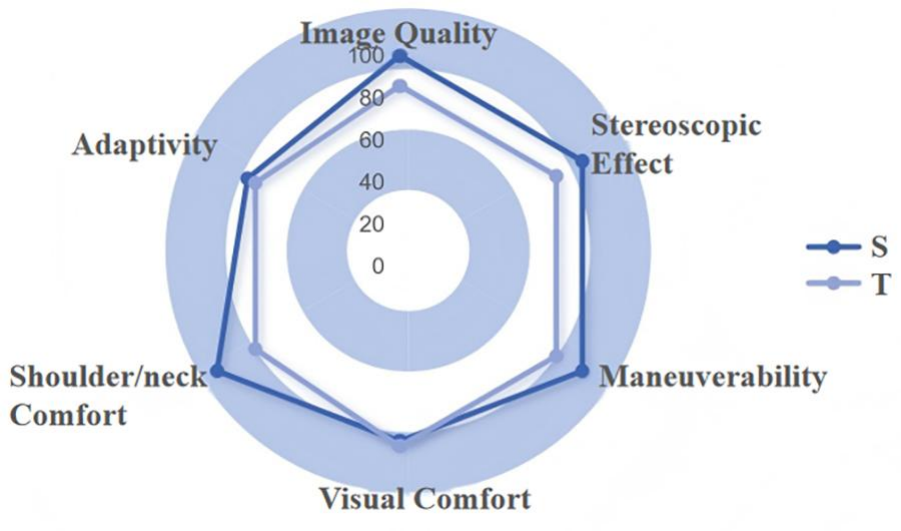
Statistically analysis of the questionnaire. There were no statistically significant differences between the surgeons (*n* = 6) and trainees (*n* = 14) in their ratings of ergonomics, stereoscopic visual effects, and system maneuverability of 4K3D-ES. S, surgeons; T, trainees.

Perhaps most telling was the surgical team's real-world experience. After hands-on use by 6 senior surgeons and 14 trainees, feedback was overwhelmingly positive: most surgeons praised the intuitive handling, participants were impressed by the superior 3D visualization, and over two-thirds noticed significantly less physical strain during procedures (Figure 3). No statistically significant differences were observed between senior surgeons and trainees in their assessments of ergonomics, stereoscopic visual effects, or system maneuverability. These findings suggest that 4K3D-ES was generally well accepted by users with different levels of surgical experience.

## Discussion

This study examined whether a 4K three-dimensional exoscope system (4K3D-ES) can be used for stapedotomy without losing the precision expected from a conventional operative microscope (OM). Stapes surgery is a demanding test for a new visualization platform. The surgeon must work around a small footplate, the incudostapedial joint, and the prosthesis loop, where a small loss of depth perception or instrument control could affect hearing outcome. Against this background, the absence of intraoperative conversion to OM in all 52 exoscopic cases supports the technical feasibility of the system in this procedure.

The clinical outcomes were broadly comparable between the two visualization methods. Total operative duration, selected procedural phases, and intraoperative blood loss did not differ significantly between groups. Hearing recovery showed the same pattern: both cohorts achieved postoperative air-bone gap reduction, and the proportion of patients reaching an ABG <15 dB was numerically higher in the 4K3D-ES group (59.6% vs. 47.7%), although this difference was not statistically significant. No 4K3D-ES-related complication was identified during follow-up. These findings do not prove superiority. They do, however, suggest that the exoscope can preserve the functional and safety profile of conventional microscopic stapedotomy.

The 4K3D-ES differs from the OM in the way it organizes the operative field. It captures the surgical image through a 3D microsurgical camera and displays it on shared stereoscopic monitors. The surgeon and assistant can therefore work from the same visual information rather than from a primary ocular view and a secondary observation port. The system also provides high color fidelity, adjustable magnification, and real-time 3D recording (6, 12). During the study period, the 4K3D-ES team used this platform in 930 otologic microsurgeries, including tympanoplasty (*n* = 452), radical mastoidectomy (*n* = 173), inner ear surgery (*n* = 262), and lateral skull base surgery (*n* = 45). None of these procedures required conversion to OM. A recent study by Onodera et al. reported that a 3D exoscope significantly shortened surgical setup time during canal wall down tympanomastoidectomy for primary middle ear cholesteatoma (13). However, because setup time was not separately recorded in our retrospective series, we could not determine whether a similar benefit exists for 4K3D-ES-assisted stapedotomy. This wider experience supports the practicality of the system beyond the stapedotomy cases analyzed here, although it should not replace procedure-specific validation.

Other visualization strategies have also been explored in stapes surgery. Endoscopic techniques provide a minimally invasive route and can expose hidden recesses well, but the single-handed working style can be limiting during delicate prosthesis placement (14, 15). Robot-assisted endoscopic systems may improve stability and precision, yet their use remains constrained by cost, equipment requirements, setup complexity, and access (16, 17). The exoscope occupies a different position. It preserves conventional bimanual microsurgical handling while adding shared stereoscopic visualization and a more flexible working posture.

Three-dimensional exoscopes have gradually moved from neurosurgery into otology and lateral skull base surgery (18–22). This transition is not simply a matter of higher image resolution. Otologic surgery requires stable depth perception, controlled illumination on reflective bone, and enough working distance to accommodate instruments within a narrow field. The system used in this study has two practical features that are especially relevant to these demands. First, its extended working distance gives the surgeon more freedom to position the camera and instruments. Second, digital image processing and stereoscopic display can maintain a detailed image while making the field visible to the whole operative team.

The ergonomic benefit was one of the clearest perceived advantages. With the OM, the surgeon must align the eyes, neck, trunk, and microscope oculars for prolonged periods. The exoscope separates visualization from ocular fixation. Surgeons can look toward a monitor and adjust their working angle without repeatedly matching their posture to the microscope (23, 24). In longer otologic or lateral skull base procedures, this flexibility may reduce neck and shoulder strain. In stapedotomy, the operation is shorter, but the same principle still matters because the critical steps require steady posture and sustained concentration.

Image handling also deserves attention. Dynamic illumination control may reduce glare from compact white mastoid bone, and noise reduction can help preserve image clarity in lower-light settings (25). These features are less prominent in a small stapedotomy field than in mastoid dissection or cochlear implantation, but they remain useful when the surgeon needs to distinguish the footplate, chorda tympani, facial nerve region, and ossicular landmarks. Earlier reports have noted that some surgeons switch back to OM during critical steps when confidence in exoscopic visualization is limited (10, 26). In our series, experienced operators completed all stapedotomy procedures under 4K3D-ES, suggesting that the visual quality was sufficient for the most delicate stages after appropriate familiarization.

Shared 3D visualization may be particularly valuable for teaching. In conventional microscopy, the assistant and trainees often receive a compromised or delayed view. With 4K3D-ES, the team observes the same stereoscopic field in real time. This can improve coordination during instrument exchange and allows trainees to follow the surgeon's depth cues during footplate fenestration and prosthesis placement (9, 27). The questionnaire results were consistent with this experience: senior surgeons and trainees both rated ergonomics, stereoscopic image quality, and maneuverability favorably, without significant differences between the two groups.

The recording function adds another practical advantage. Critical surgical scenes can be captured in high-definition 3D through a foot pedal, creating material for case review and surgical education. For trainees, the recordings preserve the spatial relationships of the oval window niche, stapes superstructure, prosthesis, and surrounding neurovascular structures more faithfully than two-dimensional video. For experienced surgeons, they also provide a way to review difficult maneuvers and refine technique across cases. Our broader clinical experience suggests that 4K3D-ES can support training from basic temporal bone anatomy to advanced lateral skull base procedures (28). Even so, adoption remains uneven. Several reports still describe exoscope use as early-stage, with some surgeons returning to OM during critical steps (29, 30). The learning curve in our experience was manageable, but it should be acknowledged rather than minimized.

### Limitations

This study has several limitations. It was retrospective and non-randomized, and group allocation was linked to system availability and surgeon practice. Surgeon-specific technique and learning effects may therefore have influenced operative time and outcomes. The study was also limited to stapedotomy, so its findings cannot be generalized automatically to all otologic or lateral skull base procedures. Most importantly for workflow assessment, setup time was not recorded as an independent endpoint. Future prospective studies should use standardized timestamps for room setup, exoscope positioning, calibration, incision-to-closure time, and step-specific surgical phases. Randomized or paired-surgeon designs would also help separate the effect of visualization technology from the effect of operator experience.

## Conclusion

In conclusion, our retrospective analysis demonstrates that 4K3D-ES visualization is safe and technically equivalent to conventional operative microscopy in stapes surgery. While these findings support the feasibility of 4K3D-ES in this specific otologic procedure, further studies are needed to evaluate its applicability across other types of otologic or lateral skull base surgeries.

## Data Availability

The data analyzed in this study is subject to the following licenses/restrictions: The datasets generated and/or analyzed during this study include clinical patient information, intraoperative measurements, and surgeon/trainee questionnaire responses. These data contain sensitive patient information and are subject to ethical and privacy regulations. Therefore, they cannot be publicly shared. Access to anonymized data supporting the findings of this study can be provided upon reasonable request to the corresponding author, and such requests require approval from the Institutional Review Board of Chinese PLA General Hospital. Requests to access these datasets should be directed to zcyent@sina.com.

## Supplementary material

Supplementary Figure 1 Viewing schematic for 4K stereoscopic pictures. **(A)** Independent left/right perspective images were delivered to corresponding eyes through two orthogonal polarizations, generating retinal disparity for depth perception; **(B,C)** Using presbyopia spectacles (+6.00D to +7.00D) directed differential image angles to each eye to achieve 3D vision effect (the person shown in the figure is a member of the research team).

## Appendix

**Table A1:** Clinical information of the enrolled patients.

Clinical characteristic	4K3D-ES		OM		*P* value
	No. of Ears	Percent	No. of Ears	Percent	
Gender					
Female	36	69.2%	67	76.1%	
Male	16	30.8%	21	23.9%	
Total	52	100%	88	100%	0.371
Ear					
Right	25	48.1%	51	58.0%	
Left	27	51.9%	37	42.0%	
Total	52	100%	88	100%	0.257
Age in yrs. (mean ± standard deviation)	38.3 ± 9.7		39.9 ± 11.1		0.388

**Table A2:** Comparison between preoperative and postoperative audiometric results.

Audiometric parameter	Exoscope (*n* = 52)	Operation Microscope (*n* = 88)	*P* value
Pre-operation			
Air conduction, dB	60.6 ± 12.0	62.0 ± 11.7	0.492
Bone conduction, dB	25.9 ± 8.1	26.4 ± 10.0	0.751
ABG	34.6 ± 9.1	35.5 ± 11.5	0.630
Post-operation			
Air conduction, dB	39.1 ± 13.2	40.5 ± 15.6	0.592
Bone conduction, dB	24.5 ± 9.5	25.6 ± 10.8	0.549
ABG	14.6 ± 8.8	14.9 ± 11.2	0.869
ABG <15 dB, (%)	59.6	47.7	0.236

Pure tone audiometry (500–1,000–2,000–4,000 Hz) included air conduction and bone conduction; ABG, air bone gap.
