# Supplementary figures and images for: Clinical validation of 4K three-dimensional exoscope system in stapedotomy: a retrospective cohort study
Source: Front Surg. 2026 Jul 3;13:1856818. doi: 10.3389/fsurg.2026.1856818 (PMC13375906; doi:10.3389/fsurg.2026.1856818)

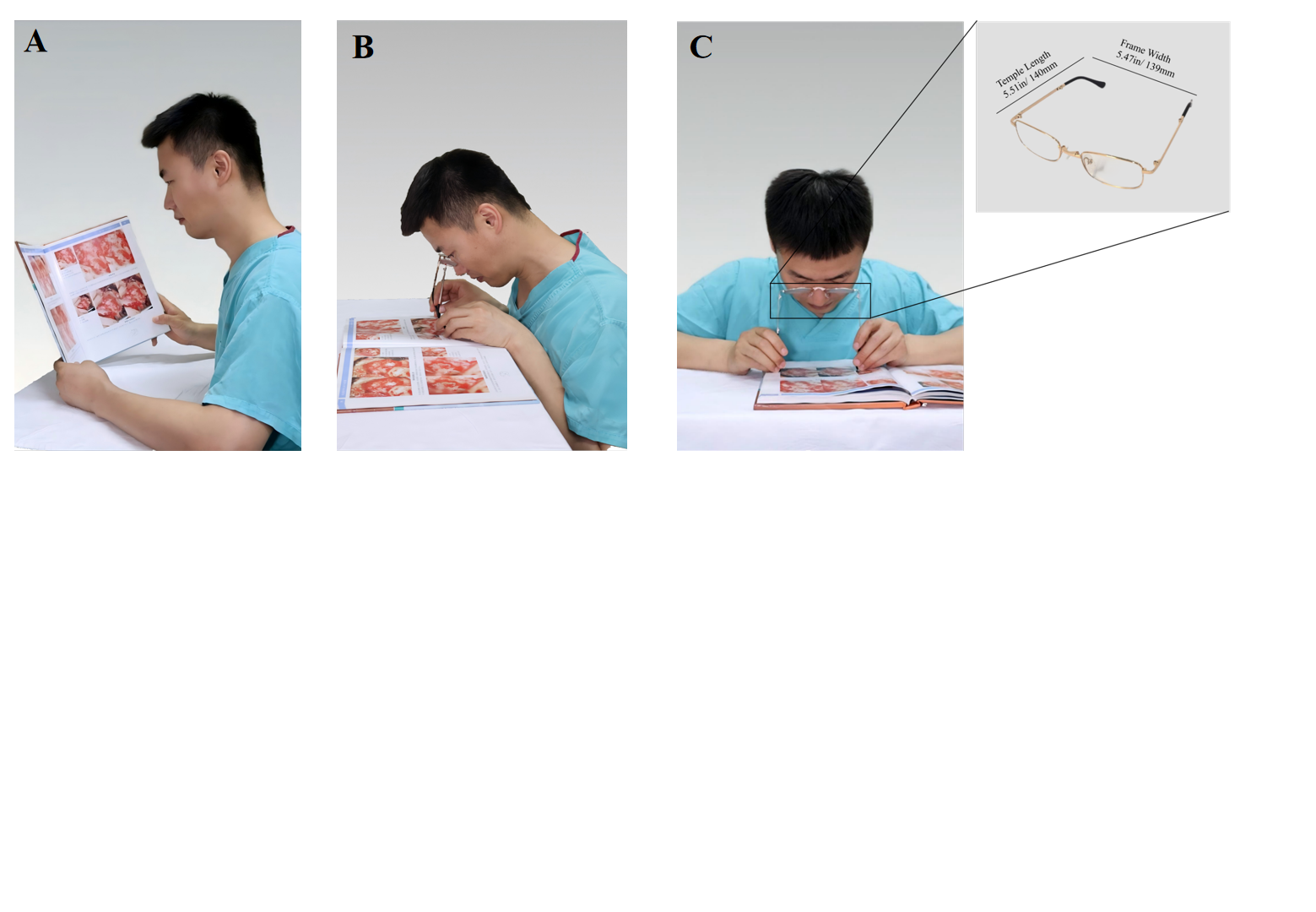

Supplement: Supplementary Figure 1 — Viewing schematic for 4K stereoscopic pictures. (A) Independent left/right perspective images were delivered to corresponding eyes through two orthogonal polarizations, generating retinal disparity for depth perception; (B,C) Using presbyopia spectacles (+6.00D to +7.00D) directed differential image angles to each eye to achieve 3D vision effect (the person shown in the figure is a member of the research team). [file Image1.tif]
